# Quality of life of the Brazilian speech therapist facing the covid-19 pandemic

**DOI:** 10.1590/2317-1782/20212021034

**Published:** 2022-01-07

**Authors:** Rubens Nasciutti Neto, Ýleris de Cássia de Arruda Mourão, Fernanda Cardoso de Oliveira Araújo

**Affiliations:** 1 Programa de Residência Multiprofissional em Endocrinologia, Hospital Estadual Geral de Goiânia Dr. Alberto Rassi – HGG/SEST - Goiânia (GO), Brasil.; 2 Departamento de Tutoria do Programa de Residência Multiprofissional em Endocrinologia, Hospital Estadual Geral de Goiânia Dr. Alberto Rassi – HGG/SEST - Goiânia (GO), Brasil.

**Keywords:** COVID-19, Quality of Life, Phonoaudiologie, Health Professionals, Brazil

## Abstract

**Purpose:**

To describe the quality of life (QoL) of Brazilian speech therapists and relate it to sociodemographic and professiographic aspects, and to their role in the COVID-19 pandemic.

**Methods:**

Population-based, cross-sectional epidemiological study. The research involved speech therapists from Brazil, who were invited to answer, online, the WHOQOL-bref form about their health and QoL, in addition to sociodemographic and professional questions.

**Results:**

609 speech therapists aged between 21 and 65 years, average age of 34 years, participated in the study. Lower scores in the physical domain of QoL were given by female speech therapists, providing outpatient care, who did not work in private clinics, acted indirectly in the pandemic, were absent from work due to psycho-emotional aspects; in the psychological domain, they were female, without a partner, working in a private clinic or elsewhere and acted directly or indirectly in the pandemic; in the social relations domain, they had no partner, worked in outpatient care, but not in private clinics; and in the environment domain, lower scores were in black and brown race/color, without a partner, attending home care, but not in a private clinic or management/administrative or other.

**Conclusion:**

Brazilian speech therapists had the lowest QoL scores in the psychological and environmental domains; their QoL is related to factors such as sex, color/race, presence of a partner, council region, service environments, direct action with COVID-19 patients and COVID-19 diagnosis.

## INTRODUCTION

On December 31, 2019, China notified the World Health Organization (WHO) of pneumonia cases of unknown etiology in the city of Wuhan and, initially, there were 44 cases. On January 7th and 12th, 2020, respectively, China disclosed the place (Wuhan Seafood Market) where the transmission of the new coronavirus to humans took place and published the genetic sequence of this microorganism. About two weeks later, there were already 282 infected in some Asian countries^(1)^. Subsequently, on March 11, 2020, the WHO declared this viral infection to be in a pandemic state^(2)^.

The disease called COVID-19 is caused by the SARS-COV-2 virus, which belongs to a family of coronaviruses similar to SARS-COV and MERS-COV, responsible for respiratory syndromes^(3)^. It is transmitted from person to person by means of the propagation of droplets (coughing or sneezing, for example), in direct contact with contaminated surfaces or by air in procedures that generate aerosols, such as orotracheal intubation and the like^(4-6)^.

The symptoms presented after contamination by the new coronavirus range from mild repercussions (fever, cough, difficulty breathing, gastrointestinal events) to potentially lethal ones (respiratory failure, septic shock). Some infected with SARS-COV-2 are asymptomatic and chronic patients are more susceptible to serious complications of COVID-19^(5,6)^.

According to the epidemiological bulletin of the Ministry of Health, until October 2020, Brazil registered 5,224,362 cases and 153,675 deaths, corresponding to almost 15% of the number of infected people around the world^(7)^. Due to the high rate of transmissibility, the recommendation is to redouble prevention measures, including the use of masks and social distancing^(8)^.

For suspected cases, health professionals are responsible for requesting laboratory tests (immunological or molecular biology - reverse transcription polymerase chain reaction - RT PCR) and following the other recommendations of the Ministry of Health^(6-8)^. Treatment is carried out according to risk stratification guiding the therapy^(8)^.

Health professionals, such as speech therapists, have a prominent role in the fight against the pandemic^(9)^. In Intensive Care Units, the continuity of speech therapy intervention is essential, especially to manage swallowing disorders. Therefore, after weaning from orotracheal intubation, when there is clinical improvement of the COVID-19 patient, the assessment/conduct of a speech therapist is requested^(10)^. Therefore, in the hospital context, the Federal Council of Speech Therapists (CFFa) and the Brazilian Intensive Care Medicine Association (AMIB) recommended to speech therapists: suspension of in-person outpatient care (elective procedures/exams), participation in training of the Committee for Control and Hospital Infection (CCHI) and an professional to care exclusively for those patients under investigation of infection or for those infected by the new coronavirus^(9,10)^.

In Brazil, Ordinance 639/2020 asked the supervisory bodies, including the CFFa, to send the list of registered people with active enrollment and defined the registration/training of health professionals in online courses, since the proper development of their skills is crucial for coping with COVID-19^(11)^.

Given all the adversities that the pandemic inflicted on the population and society in general, it is essential to address the promotion of quality of life (QL), which is conceptualized by the WHO as the way in which human beings see their place in life, culture and values intrinsic to it^(12)^. Considering aspects of mental health, a cross-sectional study recorded that most Chinese health professionals working in hospitals exclusively for COVID-19 reported manifestations such as depression, anxiety, insomnia, anguish, resulting from the fear of getting sick and/or transmitting the disease to those they live with^(13)^.

Thus, this research proposed to assess the quality of life of Brazilian speech therapists and relate it to sociodemographic and professiographic aspects, and those related to their role in the COVID-19 pandemic.

## METHODS

This is an epidemiological, cross-sectional, descriptive-analytical, quantitative study with information related to the quality of life of speech therapists facing COVID-19, recruited through social media channels (WhatsApp®, Instagram®, Facebook®). This study was approved by the Research Ethics Committee of the Dr. Alberto Rassi State General Hospital in Goiânia, Brazil, under protocol number 4.094,980.

The sample calculation was performed considering the population of 45,123 speech therapists in Brazil^(14)^, sampling error of 5%, confidence level of 95%. Thus, the calculated sample size was 381 professionals, to which 10% were added to cover possible losses and inconsistencies, totaling 420 speech therapists. Data collection took place between June and October 2020, and ended only after reaching the minimum participation proportional to the number of professionals registered in the Regional Councils of Speech Therapists.

Speech therapists of both genders who worked in the Brazilian territory during the pandemic participated in the study. Incomplete forms and those not registered in class councils in the study period were excluded. The sample selected for this study answered the form only after reading and marking the option ‘yes’ by accepting the terms of the Informed Consent Form (Resolution CNS 510/2016)^(15)^.

This research used the specific digital questionnaire for speech therapists (Google Forms®), prepared by the research team, addressing questions about their age, gender (female/male), race/color (yellow/indigenous, black, brown, white), presence of partner (yes, no), regional council (1st to 9th CFFa), work environment (outpatient, intensive care unit, palliative care, infirmary, household, private clinic, rehabilitation center, management/administrative, other location which included public service due to the low occurrence), number of work environments (one, two, three or more), acting in the fight against the pandemic (no, yes directly or indirectly), diagnosis of COVID-19 (yes, no), hospitalization due to COVID-19, absence or not from work (yes, no), reason for absence (institutional decision, chronic illness, psycho-emotional aspects, personal choice, flu-like symptoms).

The WHOQOL-bref instrument was also used to assess the QoL. This instrument has 26 questions divided into four domains: physical, psychological, social relationships and environment, abbreviated version of the WHOQOL-100^(12)^. The domain scores were calculated by inverting the response categories for questions 3, 4 and 26 so that (1=5) (2=4) (3=3) (4=2) (5=1); and considering the following syntax:

Physical = average (Questions: 3,4,10,15,16,17,18)*4.

Psychological = average (Questions: 5,6,7,11,19,26)*4.

Social relationships = average (Questions: 20,21,22)*4.

Environment = average (Questions: 8,9,12,13,14,23,24,25)*4.

The calculation above had results with a range between 4 and 20 points, and was transformed into a scale of 0-100 points with subtraction of each domain by 4 and made the product with a value of 6.25 [(domain-4)*6.25]. For this instrument, the higher the score, the better the QoL for the domain.

For quantitative analysis, the data collected by the form were extracted and organized in Microsoft Office Excel® spreadsheets (Windows, 2013). Descriptive analysis was performed, which for categorical variables is presented in absolute (n) and relative (%) frequencies.

For continuous variables, median and interquartile range (IQR - p25-p75) were used. Shapiro Wilk test was performed to verify the normality of the data and from this, non-parametric statistics were applied to compare continuous variables, specifically the Mann-Whitney test (2 groups) and Kruskal-Wallis test (more than two groups). For significant variables, Dunn's test was used for differentiation, which is presented in lowercase letters flanked by their medians in the tables.

Also, the Spearman's correlation test was performed with an estimate of the rho coefficient (ρ) which was classified as direct correlation when positive and inverse when negative; and still in very strong correlation when equal to or greater than/less than 0.9 for more or less; strong correlation when 0.7 to 0.9 positive or negative; moderate correlation when 0.5 to 0.7 positive or negative; weak correlation when 0.3 to 0.5 positive or negative; and very weak correlation when 0 to 0.3 positive or negative. The significance level used for all tests was 5%. STATA® software version 14.0 was used in this analysis.

## RESULTS

The study included 609 speech therapists with a mean age of 33.92 years (sd= 9.70; min=21; max=65 years). Most of the sample was female (n=558, 91.63%), who had a partner (n=337, 55.34%) and white race/color (n=359, 58.95%), followed by brown (n=170, 27.91%), black (n=67, 11.00%), yellow/indigenous (n=13, 2.14%). This research reached the minimum participation foreseen in the sample calculation in all Brazilian states and also in the regions of the Speech Therapy Council: 1^st^ region (64-10.51%), 2^nd^ region (133-21.84%), 3^rd^ region (44-7.22%), 4^th^ region (66-10.84%), 5^th^ region (108-17.73%), 6^th^ region (67-11.00%), 7^th^ region (52-8.54%), 8^th^ region (36-5.91%) and 9^th^ region (39-6.40%).

The number of those who registered direct or indirect action in fighting the COVID-19 pandemic was 49.26% (n=300). Most professionals were employed in two or more jobs (313-50.97%) and their work environments included: private clinic (292-47.95%), household (242-39.74%), outpatient (166-27.26%), intensive care unit (113-18.56%), rehabilitation center (86-14.12%), infirmary (83-13.63%), palliative care (63-10.34%), another location (51-8.37%) and management/administrative (31-5.09%). About two thirds of the participants were away from work, the main reasons being: institutional decision (234-38.42%), personal choice (96 - 15.75%) and COVID-19 diagnosis (60-9.85%).

**Figure 1:**
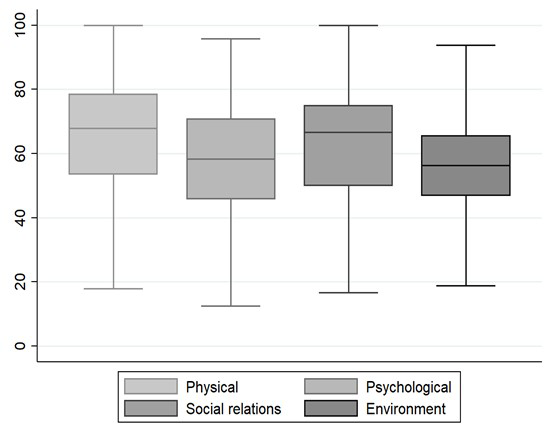
Scores in the quality of life domains of speech therapists Brazil, 2020, n=609

The median of perceived quality of life of speech therapists was 75 points (IQR=75.00-75.00) and for satisfaction with health it was 75 points (IQR=50.00-75.00). As for the domains, it was found that the median of the physical domain was 67.86 points (IQR=53.57-78.57); psychological score of 58.33 points (IQR=45.83-70.83); social relationships of 66.67 points (IQR=50.00-75.00); and environment 56.25 points (IQR=46.87-65.62) (Figure 1).

There was a significant, direct and very weak correlation of age with the psychological and social relationships domains, so that the older the age, the higher the score and, thus, the better the QoL in these domains among these participants (rho=0 .08, p=0.036 for both – Table 1). When evaluating the relationship between sociodemographic aspects and the QoL of speech therapists, it was found that for the physical domain there were higher scores, therefore better QoL in males; in the psychological domain, there were higher scores in males and in professionals who had a partner; in the social relationships domain, there was also a higher QoL for professionals with partners; and finally, for the environment domain, there was a higher QoL score for whites compared to blacks and browns, but not for yellows and indigenous people (Table 2).

**Table 1:** Correlation between age and quality of life domains of speech therapists. Brazil, 2020, n=609

	Rho (ρ)	p-value
**Physical domain**	**0.05**	**0.201**
		
**Psychological domain**	**0.08**	**0.036**
		
**Social relationships domain**	**0.08**	**0.036**
		
**Environment domain**	**0.07**	**0.082**

Spearman's correlation test with estimated rho coefficient (ρ), with 5% significance level

**Table 2:** Quality of life domains and sociodemographic issues. Brazil, 2020, n=609

	**Physical domain**	**Psychological domain**	**Social relationships domain**	**Environment domain**
**Sex**	*	^*^		
Female	67.86(53.57-75.00)	58.33(45.83-70.83)	66.67(50.00-75.00)	56.25(46.88-65.63)
Male	71.43(64.29-78.57)	66.67(58.33-75.00)	66.67(50.00-75.00)	56.25(46.88-68.75)
**Race/color**				*
Yellow / indigenous	71.43(67.86-71.43)	62.50(45.83-66.67)	75.00(58.33-75.00)	56.25(50.00-65.63)ab
White	67.86(53.57-78.57)	58.33(45.83-70.83)	66.67(50.00-75.00)	59.38(46.88-68.75)a
Black	64.29(50.00-71.43)	58.33(41.67-66.67)	58.33(41.67-75.00)	50.00(43.75-59.38)b
Brown	64.29(53.57-75.00)	58.33(45.83-70.83)	62.50(50.00-75.00)	53.13(43.75-62.50)b
**Partner**		*	*	*
Yes	67.86(53.57-78.57)	62.50(50.00-70.83)	66.67(50.00-75.00)	59.38(50.00-68.75)
No	67.86(53.57-75.00)	58.33(45.83-70.83)	58.33(50.00-75.00)	56.25(43.75-62.50)

Data in Table 2 presented as median (Interquartile range: P25 -P75)

* p<0.05, obtained by the Mann-Whitney test (2 categories of variables) and Kruskal-Wallis (more than two categories of variables). Different lowercase letters, flanked by their medians and interquartile ranges, represent differences by Dunn's test

Table 3 lists the QoL domains concerning the participants' professional questions. In the physical domain: statistically higher score recorded in private practice (67.26 points) in relation to the outpatient service and palliative care (both with 64.29 points). As for the psychological domain, the highest score for work in public service and in private practice (both with 62.50 points). On the social relationships domain, low score among those who reported working in an outpatient setting or intensive care unit or palliative care and outside the private clinic (all with 58.33 points). In the environment domain, the highest score for speech therapists from the 7th region (60.94 points), working in private clinic, rehabilitation center, management/administrative, other/public service (all with 59.38 points) and lowest score for belonging to the 9th region (50.00 points), as well as professionals at home (54.69 points). Those who reported three or more workplaces had lower scores in the physical aspects, social relationships and environment.

**Table 3:** Quality of life domains and professiographic aspects. Brazil, 2020, n=609

	**Physical domain**	**Psychological domain**	**Social relationships domain**	**Environment domain**
**Region**				*
1^st^	60.71(50.00-78.57)	58.33(41.67-70.83)	66.67(41.67-75.00)	53.13(43.75-65.63)ab
2^nd^	64.29(53.57-75.00)	62.50(45.83-70.83)	58.33(41.67-75.00)	56.25(46.88-65.63) ab
3^rd^	73.21(60.71-82.14)	56.25(45.83-70.83)	66.67(50.00-75.00)	59.38(50.00-65.63) ab
4^th^	64.29(53.57-71.43)	58.33(41.67-66.67)	66.67(50.00-75.00)	54.69(46.88-65.63) ab
5^th^	67.86(53.57-78.57)	58.33(45.83-70.83)	66.67(50.00-75.00)	56.25(40.63-65.63) ab
6^th^	67.86(57.14-78.57)	58.33(50.00-75.00)	66.67(50.00-75.00)	56.25(46.88-65.63) ab
7^th^	71.43(57.14-80.36)	58.33(50.00-72.92)	66.67(50.00-75.00)	60.94(53.13-70.31)a
8^th^	66.07(53.57-73.21)	62.50(50.00-70.83)	66.67(54.17-75.00)	54.69(46.88-64.06) ab
9^th^	67.86(57.14-75.00)	58.33(50.00-70.83)	66.67(50.00-75.00)	50.00(40.63-59.38)b
**Work environment**				
Outpatient	*		*	
No	67.86(57.14-78.57)	58.33(45.83-70.83)	66.67(50.00-75.00)	56.25(46.88-65.63)
Yes	64.29(50.00-75.00)	58.33(45.83-70.83)	58.33(41.67-75.00)	56.25(43.75-68.75)
Intensive care unit			*	
No	67.86(53.57-78.57)	58.33(45.83-70.83)	66.67(50.00-75.00)	56.25(46.88-65.63)
Yes	67.86(53.57-75.00)	58.33(45.83-66.67)	58.33(50.00-75.00)	56.25(46.88-65.63)
Palliative care	*		*	
No	67.86(53.57-78.57)	58.33(45.83-70.83)	66.67(50.00-75.00)	56.25(46.88-65.63)
Yes	64.29(53.57-71.43)	58.33(41.67-66.67)	58.33(41.67-75.00)	56.25(43.75-68.75)
Infirmary				
No	67.86(53.57-78.57)	58.33(45.83-70.83)	66.67(50.00-75.00)	56.25(46.88-65.63)
Yes	67.86(53.57-75.00)	58.33(50.00-70.83)	66.67(50.00-75.00)	56.25(50.00-65.63)
Household	*		*	*
No	67.86(57.14-78.57)	58.33(45.83-70.83)	66.67(50.00-75.00)	59.38(46.88-65.63)
Yes	66.07(50.00-75.00)	58.33(45.83-70.83)	58.33(50.00-75.00)	54.69(43.75-65.63)
Private clinic	*	*	*	*
No	64.29(53.57-75.00)	58.33(45.83-70.83)	58.33(50.00-75.00)	56.25(43.75-65.63)
Yes	67.86(57.14-78.57)	62.50(50.00-70.83)	66.67(50.00-75.00)	59.38(46.88-65.63)
Rehabilitation center				*
No	67.86(53.57-78.57)	58.33(45.83-70.83)	66.67(50.00-75.00)	56.25(46.88-65.63)
Yes	67.86(53.57-75.00)	58.33(45.83-70.83)	66.67(50.00-75.00)	59.38(43.75-65.63)
Management/administrative		*		*
No	67.86(53.57-78.57)	58.33(45.83-70.83)	66.67(50.00-75.00)	56.25(46.88-65.63)
Yes	67.86(53.57-75.00)	62.50(45.83-66.67)	58.33(41.67-66.67)	59.38(53.13-71.88)
Others	^*^	*		*
No	67.86(53.57-78.57)	58.33(45.83-70.83)	66.67(50.00-75.00)	56.25(46.88-65.63)
Yes	71.43(60.71-78.57)	62.50(54.17-75.00)	58.33(50.00-75.00)	59.38(50.00-68.75)
Nº of work environments*			*	*
One	67.86(57.14-78.57)	58.33(45.83-70.83)	66.67(50.00-75.00)	56.25(46.88-65.63)
Two	67.86(53.57-78.57)	58.33(45.83-70.83)	66.67(50.00-75.00)	56.25(46.88-65.63)
Three or more	64.29(53.57-75.00)	58.33(45.83-70.83)	58.33(45.83-75.00)	59.38(46.88-68.75)

Data in Table 3 presented as median (Interquartile range: P25 -P75)

* p<0.05, obtained by the Mann-Whitney test (2 groups) and Kruskal-Wallis (more than two groups). Different lowercase letters, flanked by their medians and interquartile ranges, represent differences by Dunn's test

Table 4 lists the QoL domains of the participants and the questions related to COVID-19. In the physical domain: statistically lower score among those who worked indirectly to fight the pandemic or who were away from work due to chronic illness, flu-like symptoms, COVID-19 diagnosis (all with 64.29 points), psycho-emotional aspects (57.14 points). As for the psychological domain, the highest score was for those who did not act against COVID-19 or who reported being absent from work due to personal choice (both with 62.50 points). In the social relationships domain, those who were dismissed for having flu-like symptoms had a reduced score (62.50 points). And in the environment domain lower scores for those who experienced absence from work (56.25 points) due to psycho-emotional aspects or had COVID-19 (both with 50.00 points).

**Table 4:** Quality of life domains of participants and issues related to COVID-19 Brazil, 2020, n=609

	**Physical domain**	**Psychological domain**	**Social relationships domain**	**Environment domain**
**Confrontation of the COVID-19 pandemic**	*	*		
Yes, directly	67.86(57.14-78.57)a	58.33(45.83-70.83)a	66.67(50.00-75.00)	56.25(46.88-65.63)
Yes, indirectly	64.29(50.00-75.00)b	58.33(45.83-66.67)a	58.33(50.00-75.00)	56.25(46.88-65.63)
No	67.86(53.57-78.57)a	62.50(45.83-70.83)b	58.33(50.00-75.00)	56.25(46.88-65.63)
**Had COVID-19**	*			*
No	67.86(53.57-78.57)	58.33(45.83-70.83)	66.67(50.00-75.00)	56.25(46.88-65.63)
Yes	64.29(46.43-75.00)	58.33(41.67-70.83)	66.67(50.00-75.00)	50.00(40.63-68.75)
COVID-19 diagnosis, no hospitalization	*	*		
No	67.86(53.57-78.57)	58.33(45.83-70.83)	66.67(50.00-75.00)	56.25(46.88-65.63)
Yes	64.29(50.00-78.57)	58.33(41.67-70.83)	66.67(50.00-66.67)	50.00(37.50-68.75)
COVID-19 diagnosis, hospitalization				
No	67.86(53.57-78.57)	58.33(45.83-70.83)	66.67(50.00-75.00)	56.25(46.88-65.63)
Yes	67.86(53.57-78.57)	58.33(45.83-70.83)	66.67(50.00-75.00)	56.25(46.88-65.63)
Absence from work *				*
No	67.86(57.14-78.57)	58.33(45.83-70.83)	66.67(50.00-75.00)	59.38(46.88-65.63)
Yes	66.07(53.57-75.00)	58.33(45.83-70.83)	66.67(50.00-75.00)	56.25(46.88-65.63)
**Reason for absence**				
Institutional decision				
No	64.29(53.57-75.00)	58.33(45.83-70.83)	58.33(50.00-75.00)	56.25(46.88-65.63)
Yes	64.29(53.57-75.00)	58.33(45.83-70.83)	58.33(50.00-75.00)	56.25(46.88-65.63)
Chronic disease	*			
No	67.86(53.57-78.57)	58.33(45.83-70.83)	66.67(50.00-75.00)	56.25(46.88-65.63)
Yes	64.29(46.43-78.57)	58.33(41.67-70.83)	66.67(50.00-75.00)	56.25(46.88-65.63)
Psycho-emotional aspects *				*
No	67.86(53.57-78.57)	58.33(45.83-70.83)	66.67(50.00-75.00)	56.25(46.88-65.63)
Yes	57.14(35.71-71.43)	58.33(37.50-66.67)	66.67(41.67-75.00)	50.00(43.75-65.63)
Personal choice	*	*		
No	67.86(53.57-78.57)	58.33(45.83-70.83)	66.67(50.00-75.00)	56.25(46.88-65.63)
Yes	69.64(60.71-78.57)	62.50(50.00-70.83)	66.67(50.00-75.00)	56.25(46.88-65.63)
Flu-like symptoms	*		*	
No	67.86(53.57-78.57)	58.33(45.83-70.83)	66.67(50.00-75.00)	56.25(46.88-65.63)
Yes	64.29(50.00-78.57)	58.33(45.83-70.83)	62.50(41.67-75.00)	56.25(43.75-64.06)
COVID-19 diagnosis, no hospitalization ^*^				*
No	67.86(53.57-78.57)	58.33(45.83-70.83)	66.67(50.00-75.00)	56.25(46.88-65.63)
Yes	64.29(50.00-78.57)	58.33(41.67-70.83)	66.67(50.00-66.67)	50.00(37.50-68.75)
COVID-19 diagnosis, hospitalization				
No	67.86(53.57-78.57)	58.33(45.83-70.83)	66.67(50.00-75.00)	56.25(46.88-65.63)
Yes	67.86(53.57-78.57)	58.33(45.83-70.83)	66.67(50.00-75.00)	56.25(46.88-65.63)

Data in Table 4 presented as median (Interquartile range: P25-P75)

* p<0.05, obtained by the Mann-Whitney test (2 groups) and Kruskal-Wallis (more than 2 groups). Different lowercase letters, flanked by their medians and interquartile ranges, represent differences by Dunn's test

## DISCUSSION

This research proposes to assess the QoL of Brazilian speech therapists in the pandemic context of COVID-19. Some research on occupational safety, mental health and QoL of health professionals during the COVID-19 pandemic could be found^(13,16)^.

An Australian study^(17)^ pointed out a higher risk of psychological distress in young health professionals during viral outbreaks, due to restrictions imposed by quarantine, social stigma and impaired sleep, corroborating the present study in which the elderly speech therapist presented higher QoL scores for the psychological and social relationships domains, showing that with advancing age, the social support network of these professionals can improve their QoL.

Females had worse scores compared to males in physical and psychological aspects, corroborating Brazilian research^(18)^ that described reduced QoL scores in female medical students, in the same WHOQOL-bref domains, before the pandemic.

Most Brazilian speech therapists participating in this study are women and recent literature^(19)^ has shown a higher prevalence of stress, anxiety and depression symptoms in the female population during the COVID-19 pandemic, in addition to their emotional vulnerability linked to hormonal changes during the premenstrual period, pre- and postpartum and menopause, as well as gender inequalities, which strengthen work overload, reflecting on QoL.

Brown and black speech therapists had lower QoL scores for the environment domain. According to a research published by the Brazilian Institute of Geography and Statistics, people with black or brown skin have lower income even in a similar position and limited reach to a higher position compared to whites^(20)^, and it is assumed that in the pandemic scenario these discrepancies affect even more the QoL .

As for marital status, speech therapists who are married or in a stable relationship had higher QoL scores for the social, psychological and environmental domains compared to those who reported having no partner. Studies attest the importance of the support network^(17,19)^ during the pandemic and show the reflection of social coexistence on QoL, as well as possible greater economic security when one can count on the partner to manage household expenses^(21)^.

For the environment domain, speech therapists from the 7^th^ region of the Council of Speech Therapy (Rio Grande do Sul state) had the best QoL score and the lowest score came from the 9^th^ region (states of Acre, Amapá, Amazonas, Pará, Rondônia, Roraima). There may be a relationship with socioeconomic inequalities (in infrastructure, income distribution, basic sanitation)^(20)^ and with an average difference of 52% in the mortality rate from SARS-COV-2 between the South and North regions of Brazil (44.1 and 84.4 deaths for every 100 thousand inhabitants, respectively)^(7)^, during this research.

Regarding the work environment, the QoL scores of speech therapists in private clinics, rehabilitation centers, management/administrative were higher than those who work in an outpatient clinic, in the household, intensive care unit and palliative care, possibly, as shown by other studies^(16,22)^ due to the intense service, the low availability of personal protective equipment and the constant feeling of insecurity experienced by professionals in hospital and/or home care, as well as the suspension of the in-person outpatient service^(10)^ linked to absence from work or teleservice, both with a likely salary impact.

Based on the data found and the realities experienced by speech therapists during the first period of the pandemic, an increase in the social support network is suggested, reflecting in the cordial and ethical relationship among colleagues, as well as the search for a feeding routine, physical exercise, regular sleep periods, therapies, meditations and relaxation techniques as possibilities in coping with the COVID-19 pandemic.

A limitation in this study was the elaboration of more assertive questions regarding professional aspects. Strategies such as request from the council region and comparison with the state of residence were used to restrict answers only to speech therapists, single filling for each participant and obligatoriness in the relevant and complementary questions, in an attempt to reduce uncertainties in the answers.

## CONCLUSION

Brazilian speech therapists had lower QoL scores in the psychological and environmental domains in the context of the COVID-19 pandemic. Lower scores in the physical domain of QOL were given by female speech therapists, who provided outpatient care, did not work in private clinics, acted indirectly in the pandemic, who left work and whose reason for leaving were psycho-emotional aspects. As for the psychological domain of QoL, females, the absence of a partner, professionals who worked in a private clinic or elsewhere and who act directly or indirectly in the pandemic had lower scores compared to their peers.

Assessing the social relationships domain, the lowest scores were found in professionals who did not have a partner, in those who performed outpatient care but did not work in private clinics. And finally, for the environment domain, lower scores were found in the black and brown race/color, in those without a partner, in those who provided home care, but not in those who attended in private clinics o r who worked in management/administration or another location. Thus, it is concluded that the QoL of speech therapists during the pandemic is related to sociodemographic, professiographic and COVID-19 factors.

Brazilian speech therapists work on the front lines to contain the spread of the new coronavirus and take care of sick people, while putting their own lives at risk. Possibly the concern for others overlaps the concern for themselves. Therefore, knowing that their families are safe, and that friends and society value their work is essential for them to be able to face with courage and hope the difficult task in which they are engaged. Studies show that the balanced use of social networks, personal care and empathy are ways to mitigate the impacts on the QoL.

Finally, further studies in this area are suggested in order to point out, associate and compare the quality of life of health professionals in the years following the new coronavirus pandemic.
