# Supplementary material for: Quality of life of the Brazilian speech therapist facing the covid-19 pandemic
Source: Codas. 2022 Jan 7;34(3):e20210034. doi: 10.1590/2317-1782/20212021034 (PMC9769421; doi:10.1590/2317-1782/20212021034)
Supplement: Translation

# Translation: Qualidade de vida do fonoaudiólogo brasileiro frente à pandemia da COVID-19

**Authors:** Rubens Nasciutti Neto, Ýleris de Cássia de Arruda Mourão, Fernanda Cardoso de Oliveira Araújo

**Affiliations:** 1 Programa de Residência Multiprofissional em Endocrinologia, Hospital Estadual Geral de Goiânia Dr. Alberto Rassi – HGG/SEST - Goiânia (GO), Brasil.; 2 Departamento de Tutoria do Programa de Residência Multiprofissional em Endocrinologia, Hospital Estadual Geral de Goiânia Dr. Alberto Rassi – HGG/SEST - Goiânia (GO), Brasil.

**Keywords:** COVID-19, Qualidade de Vida, Fonoaudiologia, Profissionais de Saúde, Brasil

## Abstract

**Objetivo:**

Descrever a qualidade de vida (QV) do fonoaudiólogo brasileiro e relacionar com aspectos sociodemográficos, profissiográficos e relativos à atuação na pandemia de COVID-19.

**Método:**

Estudo epidemiológico de base populacional, transversal. A pesquisa envolveu fonoaudiólogos do Brasil, que foram convidados a responder, on-line, o formulário WHOQOL-bref sobre a sua saúde e QV, além de questões sociodemográficas e profissiográficas.

**Resultados:**

Participaram 609 fonoaudiólogos na faixa etária entre 21 e 65 anos e média de 34 anos. Menores pontuações no domínio físico da QV foram dadas por fonoaudiólogos do sexo feminino, que faziam atendimento ambulatorial, não trabalhavam em clínica particular, atuavam de forma indireta na pandemia, se afastaram do trabalho e cujo motivo de afastamento foram aspectos psicoemocionais; no domínio psicológico, sexo feminino, ausência de companheiro, que trabalhavam em clínica particular ou outro local e que atuaram de forma direta ou indireta na pandemia; no domínio relações sociais, ausência de companheiro, em atendimento ambulatorial, mas não em clínicas particulares; e no domínio meio ambiente, menores escores na raça/cor preta e parda, nos com ausência de companheiro, que faziam atendimento domiciliar, mas não em clínica particular ou gestão/administrativo ou outro.

**Conclusão:**

O fonoaudiólogo brasileiro apresentou mais baixos escores de QV nos domínios psicológico e meio ambiente e verificou-se que a sua QV está relacionada a fatores como sexo, cor/raça, presença de companheiro, região do conselho, ambientes de atendimento, atuação direta com pacientes de COVID e diagnóstico de COVID-19.

## INTRODUÇÃO

Em 31 de dezembro de 2019, a China notificou à Organização Mundial da Saúde (OMS) os casos de pneumonias com etiologia desconhecida na cidade de Wuhan e, inicialmente, eram 44 casos. Nos dias 07 e 12 de janeiro de 2020, respectivamente, a China divulgou o local (mercado de frutos do mar de Wuhan) onde ocorreu a transmissão do novo coronavírus a seres humanos e publicou a sequência genética deste microrganismo. Cerca de duas semanas depois já existiam 282 infectados em alguns países asiáticos^(1)^. Posteriormente, em 11 de março de 2020, a OMS declarou estado de pandemia à esta infecção viral^(2)^.

A doença denominada COVID-19 é causada pelo vírus SARS-COV-2 que pertence a uma família de coronavírus semelhante ao SARS-COV e MERS-COV, responsáveis por causar síndromes respiratórias^(3)^. A forma de transmissão é de pessoa para pessoa por meio da propagação de gotículas (tosse ou espirro, por exemplo), no contato direto com superfícies contaminadas ou por via aérea em procedimentos que geram aerossóis, como intubação orotraqueal e similares^(4-6)^.

A sintomatologia apresentada após a contaminação pelo novo coronavírus varia entre repercussões leves (febre, tosse, dificuldade para respirar, ocorrências gastrointestinais) à potencialmente letais (falência respiratória, choque séptico). Alguns infectados pelo SARS-COV-2 são assintomáticos e os doentes crônicos têm maior susceptibilidade às complicações graves da COVID-19^(5,6)^.

No boletim epidemiológico do Ministério da Saúde, o Brasil registrou até outubro de 2020, o total de 5.224.362 casos e 153.675 óbitos, correspondente a quase 15% do quantitativo de infectados ao redor do mundo^(7)^. Devido ao alto índice de transmissibilidade, a recomendação é redobrar as medidas de prevenção, incluindo o uso de máscaras e distanciamento social^(8)^.

Para casos suspeitos, o profissional de saúde é responsável por solicitar exames laboratoriais (imunológico ou de biologia molecular- reverse transcription polymerase chain reaction - RT PCR) e seguir as demais recomendações do Ministério da Saúde^(6-8)^. O tratamento é feito segundo a estratificação de risco para nortear a terapêutica^(8)^.

O profissional de saúde, como o fonoaudiólogo, tem papel de destaque no combate à pandemia^(9)^. Nas Unidades de Terapia Intensiva a continuidade da intervenção fonoaudiológica é indispensável, especialmente para manejar os distúrbios da deglutição. Portanto, após o desmame da intubação orotraqueal, quando da melhora clínica do usuário com COVID-19, é solicitada a avaliação/conduta de profissional da Fonoaudiologia^(10)^. Diante disso, no contexto hospitalar, o Conselho Federal de Fonoaudiologia (CFFa) e a Associação de Medicina Intensiva Brasileira (AMIB) recomendaram aos fonoaudiólogos: suspensão da assistência ambulatorial presencial (procedimentos/exames eletivos), participação nos treinamentos da Seção de Comissão de Controle e Infecção Hospitalar (CCIH) e que houvesse um profissional exclusivo para atendimento daqueles pacientes em investigação de infecção ou para aqueles infectados pelo novo coronavírus^(9,10)^.

No Brasil, a Portaria 639/2020 solicitou aos órgãos fiscalizadores, entre estes o CFFa, o envio da lista de registrados com inscrição ativa e definiu o cadastro/capacitação dos profissionais de saúde, em cursos à distância, visto que o desenvolvimento adequado de suas competências é crucial para o enfrentamento da COVID-19^(11)^.

Isto posto e mediante todas as adversidades que a pandemia infligiu à população e sociedade de um modo geral, é indispensável abordar a promoção da qualidade de vida (QV), que é conceituada pela OMS como a maneira pela qual o ser humano enxerga seu lugar na vida, na cultura e nos valores intrínsecos a ele^(12)^. Considerando aspectos da saúde mental um estudo transversal registrou que a maioria dos profissionais de saúde chineses lotados em hospitais exclusivos para COVID-19, relataram manifestações como depressão, ansiedade, insônia, angústia, decorrentes do receio de adoecer e/ou transmitir a doença para aqueles de sua convivência^(13)^.

Sendo assim, a presente pesquisa propôs avaliar a qualidade de vida do fonoaudiólogo brasileiro e relacionar com os aspectos sociodemográficos, profissiográficos e relativos à atuação na pandemia de COVID-19.

## MÉTODO

Trata-se de um estudo epidemiológico de base populacional, transversal, descritivo-analítico, de caráter quantitativo, com informações relacionados a qualidade de vida do fonoaudiólogo frente a COVID-19, recrutados por meio de canais de mídia social (*WhatsApp*®, *Instagram*®, *Facebook*®). Esse estudo foi aprovado pelo Comitê de Ética em Pesquisa do Hospital Estadual Geral de Goiânia Dr. Alberto Rassi, sob o parecer número 4.094.980.

O cálculo amostral foi realizado considerando a população de 45.123 profissionais fonoaudiólogos no Brasil^(14)^, erro amostral de 5%, nível de confiança de 95%. Dessa forma, o tamanho amostral calculado foi de 381 profissionais, aos quais foram adicionados 10% para cobertura de possíveis perdas e inconsistências, totalizando 420 fonoaudiólogos. A coleta ocorreu entre junho e outubro de 2020, com encerramento somente após ter sido alcançada a participação mínima proporcional à quantidade desses profissionais registrados nos Conselhos Regionais de Fonoaudiologia.

Participaram do estudo fonoaudiólogos de ambos os sexos que atuavam no território nacional durante a pandemia, tendo sido excluídos os formulários incompletos e aqueles sem registro em conselho de classe no período estabelecido. A amostra selecionada para este estudo respondeu o formulário, apenas após a leitura e marcação da opção sim, em Declaração de aceitação e concordância com as informações do Termo de Consentimento Livre Esclarecido (Resolução CNS 510/2016)^(15)^.

Esta pesquisa utilizou o questionário digital específico para Fonoaudiólogos (via Google Forms®), elaborado pela equipe de pesquisa, abordando questionamentos sobre sua idade, sexo (feminino/masculino), raça/cor (amarela/indígena, preta, parda, branca), presença de companheiro (sim, não), regional de atuação (1ª a 9ª CFFa), ambiente de trabalho (ambulatorial, unidade de terapia intensiva, cuidados paliativos, enfermaria, domiciliar, clínica particular, centro de reabilitação, gestão/admnistrativo, outro local que incluiu serviço público devido à baixa ocorrência), número de ambientes de trabalho (um, dois, três ou mais), atuação no enfrentamento da pandemia (Não, Sim de forma direta ou indireta), diagnóstico de COVID- 19 (sim, não), internação devido ao COVID-19, afastamento ou não do local de trabalho (sim, não), motivo desse afastamento (decisão institucional, doença crônica, aspectos psicoemocionais, escolha pessoal, sintomas gripais).

O instrumento WHOQOL-*bref* também foi utilizado para avaliar a QV. Esse instrumento conta com 26 questões divididas em quatro domínios: físico, psicológico, relações sociais e meio ambiente, versão abreviada do WHOQOL-100^(12)^. Os escores dos domínios foram calculados invertendo as categorias de resposta para as questões 3, 4 e 26 de modo que (1=5) (2=4) (3=3) (4=2) (5=1); e considerando a seguinte sintaxe:

Físico= média(Questões: 3,4,10,15,16,17,18)*4.

Psicológico= média(Questões: 5,6,7,11,19,26)*4.

Relações sociais = média(Questões: 20,21,22)*4.

Meio ambiente= média(Questões: 8,9,12,13,14,23,24,25)*4.

O cálculo acima teve resultados com intervalo entre 4 e 20 pontos, e foi transformado em uma escala de 0-100 pontos com subtração de cada um dos domínios por 4 e feito o produto com o valor de 6,25 [(domínio-4)*6,25]. Para esse instrumento, quanto maior o escore, melhor foi a QV para o domínio.

Para análise quantitativa, os dados coletados pelo formulário foram extraídos e organizados em planilhas do Microsoft Office Excel ® (Windows, 2013). Foi realizada análise descritiva que para as variáveis categóricas está apresentada em frequências absolutas (n) e relativas (%).

Para as variáveis contínuas, utilizou-se mediana e intervalo interquartil (IIQ - p25-p75). Foi realizada teste de Shapiro Wilk para averiguar a normalidade dos dados e a partir disto, foi aplicada estatística não paramétrica para comparação das variáveis contínuas, especificamente o teste de Mann-Whitney (2 grupos) e Kruskal-Wallis (mais de dois grupos). Para as variáveis significantes foi usado teste de Dunn, para diferenciação que está apresentado em letras minúsculas ladeadas de suas medianas nas tabelas.

Foi realizado ainda teste de correlação de Spearman com estimativa do coeficiente rho (ρ) que foi classificado em correlação direta quando positivo e inversa quando negativa; e ainda em correlação muito forte quando igual ou maior/menor que 0,9 para mais ou para menos; correlação forte quando de 0,7 a 0,9 positivo ou negativo; correlação moderada quando de 0,5 a 0,7 positivo ou negativo; correlação fraca quando de 0,3 a 0,5 positivo ou negativo; e correlação muito fraca quando de 0 a 0,3 positivo ou negativo. O nível de significância utilizado para todos os testes foi de 5%. Foi utilizado o software STATA® versão 14.0 nesta análise.

## RESULTADOS

Participaram do estudo 609 fonoaudiólogos com média de idade de 33,92 anos (dp= 9,70; min=21; max=65 anos). A maioria da amostra foi do sexo feminino (n=558, 91,63%), que tinha companheiro (n= 337, 55,34%) e da raça/cor branca (n=359, 58,95%), seguida por parda (n=170, 27,91%), preta (n=67, 11,00%), amarela/indígena (n-13, 2,14%). Esta pesquisa alcançou a participação mínima prevista no cálculo amostral em todos os estados brasileiros e também nas regiões do Conselho de Fonoaudiologia: 1ª região (64-10,51%), 2ª região (133-21,84%), 3ª região (44-7,22%), 4ª região (66-10,84%), 5ª região (108-17,73%), 6ª região (67-11,00%), 7ª região (52-8,54%), 8ª região (36-5,91%) e 9ª região (39-6,40%).

O quantitativo dos que registraram atuação direta ou indireta no enfrentamento à pandemia de COVID-19 foi 49,26% (n=300). A maioria dos profissionais tinha ocupação em dois ou mais empregos (313 - 50,97%) e seus ambientes laborais incluíram: clínica particular (292-47,95%), domiciliar (242-39,74%), ambulatorial (166 -27,26%), unidade de terapia intensiva (113-18,56%), centro de reabilitação (86-14,12%), enfermaria (83-13,63%), cuidados paliativos (63-10,34%), outro local (51-8,37%) e gestão/ administrativo (31-5,09%). Cerca de 2/3 dos participantes estavam afastados do trabalho, sendo os principais motivos: decisão institucional (234-38,42%), escolha pessoal (96-15,75%) e diagnóstico de COVID-19 (60-9,85%).

A mediana de percepção da qualidade de vida dos fonoaudiólogos foi de 75 pontos (IIQ=75,00-75,00) e para satisfação com a saúde foi de 75 pontos (IIQ=50,00-75,00). Quanto aos domínios verificou-se que a mediana do domínio físico foi de 67,86 pontos (IIQ=53,57-78,57); psicológico de 58,33 pontos (IIQ=45,83-70,83); relações sociais de 66,67 pontos (IIQ=50,00-75,00); e de meio ambiente 56,25 pontos (IIQ=46,87- 65,62) (Figura 1).

**Figura 1:** Escores dos domínios de qualidade de vida de fonoaudiólogos. Brasil, 2020, n=609

Verificou-se que houve uma correlação significativa, direta e muito fraca da idade com os domínios psicológico e de relações sociais, de modo que quanto maior a idade, maior o escore e, assim, melhor a QV nesses domínios desses participantes (rho=0,08, p=0,036 para ambos – Tabela 1). Ao avaliar a relação entre os aspectos sociodemográficos e a QV de fonoaudiólogos, verificou-se que para o domínio físico houve maiores escores, portanto melhor QV no sexo masculino; no domínio psicológico, houve maiores escores no sexo masculino e nos profissionais que tinham companheiro; no domínio relações sociais, houve maior QV também para os profissionais com companheiros; e por fim, para o domínio meio ambiente, houve maior escore de QV para os de raça/cor branca em comparação aos pretos e pardos, mas não em relação a amarelos e indígenas (Tabela 2).

**Tabela 1:** Correlação entre idade e domínios de Qualidade de vida de fonoaudiólogos. Brasil, 2020, n=609

	Rho (ρ)	p-valor
Domínio físico	0,05	0,201
		
Domínio psicológico	0,08	0,036
		
Domínio relações sociais	0,08	0,036
		
Domínio meio ambiente	0,07	0,082

Teste de correlação de Spearman com estimativa de coeficiente rho (ρ), com 5% de nível de significância

**Tabela 2:** Domínios de qualidade de vida e as questões sociodemográficas. Brasil, 2020, n=609

	**Domínio físico**	**Domínio psicológico**	**Domínio relações sociais**	**Domínio meio ambiente**
**Sexo**	*	*		
Feminino	67,86(53,57-75,00)	58,33(45,83-70,83)	66,67(50,00-75,00)	56,25(46,88-65,63)
Masculino	71,43(64,29-78,57)	66,67(58,33-75,00)	66,67(50,00-75,00)	56,25(46,88-68,75)
**Raça/cor**				*
Amarela/ indígena	71,43(67,86-71,43)	62,50(45,83-66,67)	75,00(58,33-75,00)	56,25(50,00-65,63)ab
Branca	67,86(53,57-78,57)	58,33(45,83-70,83)	66,67(50,00-75,00)	59,38(46,88-68,75)a
Preta	64,29(50,00-71,43)	58,33(41,67-66,67)	58,33(41,67-75,00)	50,00(43,75-59,38)b
Parda	64,29(53,57-75,00)	58,33(45,83-70,83)	62,50(50,00-75,00)	53,13(43,75-62,50)b
**Companheiro**		*	*	*
Sim	67,86(53,57-78,57)	62,50(50,00-70,83)	66,67(50,00-75,00)	59,38(50,00-68,75)
Não	67,86(53,57-75,00)	58,33(45,83-70,83)	58,33(50,00-75,00)	56,25(43,75-62,50)

Dados da Tabela 2 apresentados em mediana (Intervalo interquartil: P25 -P75). *p<0,05, obtido pelo teste de Mann-Whitney (2 categorias da variável) e Kruskal-Wallis (mais de duas categorias das variáveis). Letras minúsculas diferentes, ladeadas de suas medianas e intervalos interquartis, representam diferenças pelo teste de Dunn

A Tabela 3 relaciona os domínios de QV às questões profissiográficas dos participantes. No domínio físico: pontuação estatisticamente superior registrada na atuação em clínica particular (67,26 pontos) em relação ao serviço ambulatorial e cuidado paliativo (ambos com 64,29 pontos). Quanto ao domínio psicológico, maior escore para os labores em serviço público e em clínica particular (ambos com 62,50 pontos). Sobre o domínio relações sociais, pontuação reduzida entre os que referiram trabalho em ambiente ambulatorial ou unidade de terapia intensiva ou cuidado paliativo e fora da clínica particular (todos com 58,33 pontos). No domínio meio ambiente, maior pontuação para os fonoaudiólogos da 7ª região (60,94 pontos), atuantes em clínica particular, centro de reabilitação, gestão/ administrativo, outro/ serviço público (todos com 59,38 pontos) e menor escore para os pertencentes à 9ª região (50,00 pontos) bem como profissionais em domicílio (54,69 pontos). Os que relataram três ou mais locais de trabalho apresentaram escores inferiores nos aspectos físico, relações sociais e meio ambiente.

**Tabela 3:** Domínios de qualidade de vida e as questões profissiográficas. Brasil, 2020, n=609

	**Domínio físico**	**Domínio psicológico**	**Domínio relações sociais**	**Domínio meio ambiente**
**Região**				*
1ª	60,71(50,00-78,57)	58,33(41,67-70,83)	66,67(41,67-75,00)	53,13(43,75-65,63)ab
2ª	64,29(53,57-75,00)	62,50(45,83-70,83)	58,33(41,67-75,00)	56,25(46,88-65,63) ab
3ª	73,21(60,71-82,14)	56,25(45,83-70,83)	66,67(50,00-75,00)	59,38(50,00-65,63) ab
4ª	64,29(53,57-71,43)	58,33(41,67-66,67)	66,67(50,00-75,00)	54,69(46,88-65,63) ab
5ª	67,86(53,57-78,57)	58,33(45,83-70,83)	66,67(50,00-75,00)	56,25(40,63-65,63) ab
6ª	67,86(57,14-78,57)	58,33(50,00-75,00)	66,67(50,00-75,00)	56,25(46,88-65,63) ab
7ª	71,43(57,14-80,36)	58,33(50,00-72,92)	66,67(50,00-75,00)	60,94(53,13-70,31)a
8ª	66,07(53,57-73,21)	62,50(50,00-70,83)	66,67(54,17-75,00)	54,69(46,88-64,06) ab
9ª	67,86(57,14-75,00)	58,33(50,00-70,83)	66,67(50,00-75,00)	50,00(40,63-59,38)b
**Ambiente de trabalho**				
Ambulatorial	*		*	
Não	67,86(57,14-78,57)	58,33(45,83-70,83)	66,67(50,00-75,00)	56,25(46,88-65,63)
Sim	64,29(50,00-75,00)	58,33(45,83-70,83)	58,33(41,67-75,00)	56,25(43,75-68,75)
Unidade de terapia intensiva			*	
Não	67,86(53,57-78,57)	58,33(45,83-70,83)	66,67(50,00-75,00)	56,25(46,88-65,63)
Sim	67,86(53,57-75,00)	58,33(45,83-66,67)	58,33(50,00-75,00)	56,25(46,88-65,63)
Cuidados paliativos	*		*	
Não	67,86(53,57-78,57)	58,33(45,83-70,83)	66,67(50,00-75,00)	56,25(46,88-65,63)
Sim	64,29(53,57-71,43)	58,33(41,67-66,67)	58,33(41,67-75,00)	56,25(43,75-68,75)
Enfermaria				
Não	67,86(53,57-78,57)	58,33(45,83-70,83)	66,67(50,00-75,00)	56,25(46,88-65,63)
Sim	67,86(53,57-75,00)	58,33(50,00-70,83)	66,67(50,00-75,00)	56,25(50,00-65,63)
Domiciliar	*		*	*
Não	67,86(57,14-78,57)	58,33(45,83-70,83)	66,67(50,00-75,00)	59,38(46,88-65,63)
Sim	66,07(50,00-75,00)	58,33(45,83-70,83)	58,33(50,00-75,00)	54,69(43,75-65,63)
Clínica particular	*	*	*	*
Não	64,29(53,57-75,00)	58,33(45,83-70,83)	58,33(50,00-75,00)	56,25(43,75-65,63)
Sim	67,86(57,14-78,57)	62,50(50,00-70,83)	66,67(50,00-75,00)	59,38(46,88-65,63)
Centro de reabilitação				*
Não	67,86(53,57-78,57)	58,33(45,83-70,83)	66,67(50,00-75,00)	56,25(46,88-65,63)
Sim	67,86(53,57-75,00)	58,33(45,83-70,83)	66,67(50,00-75,00)	59,38(43,75-65,63)
Gestão/administrativo		*		*
Não	67,86(53,57-78,57)	58,33(45,83-70,83)	66,67(50,00-75,00)	56,25(46,88-65,63)
Sim	67,86(53,57-75,00)	62,50(45,83-66,67)	58,33(41,67-66,67)	59,38(53,13-71,88)
Outro	*	*		*
Não	67,86(53,57-78,57)	58,33(45,83-70,83)	66,67(50,00-75,00)	56,25(46,88-65,63)
Sim	71,43(60,71-78,57)	62,50(54,17-75,00)	58,33(50,00-75,00)	59,38(50,00-68,75)
Nº ambientes de trabalho *			*	*
Um	67,86(57,14-78,57)	58,33(45,83-70,83)	66,67(50,00-75,00)	56,25(46,88-65,63)
Dois	67,86(53,57-78,57)	58,33(45,83-70,83)	66,67(50,00-75,00)	56,25(46,88-65,63)
Três ou mais	64,29(53,57-75,00)	58,33(45,83-70,83)	58,33(45,83-75,00)	59,38(46,88-68,75)

Dados da Tabela 3 apresentados em mediana (Intervalo interquartil: P25 -P75)

* p<0,05, obtido pelo teste de Mann-Whitney (2 grupos) e Kruskal-Wallis (mais de dois grupos). Letras minúsculas diferentes, ladeadas de suas medianas e intervalos interquartis, representam diferenças pelo teste de Dunn

A Tabela 4 relaciona os domínios de QV dos participantes e as questões relativas à COVID-19. No domínio físico: pontuação estatisticamente menor entre os que atuavam indiretamente no combate à pandemia ou que ficaram afastados do trabalho devido à doença crônica, sintomas gripais, diagnóstico de COVID-19 (todos com 64,29 pontos), aspectos psicoemocionais (57,14 pontos). Quanto ao domínio psicológico, maior escore para os que não atuaram contra a COVID-19 ou que relataram afastamento do trabalho por escolha pessoal (ambos com 62,50 pontos). Sobre o domínio relações sociais, tiveram pontuação reduzida os que foram afastados por apresentarem sintomas gripais (62,50 pontos). E no domínio meio ambiente menores escores para os que vivenciaram o afastamento do trabalho (56,25 pontos) em decorrência de aspectos psicoemocionais ou tiveram COVID-19 (ambos com 50,00 pontos).

**Tabela 4:** Domínios de qualidade de vida dos participantes e as questões relativas à COVID-19. Brasil, 2020, n=609

	**Domínio físico**	**Domínio psicológico**	**Domínio relações sociais**	**Domínio meio ambiente**
**Atuação enfrentamento** **da pandemia de COVID**	*	*		
Sim, de forma direta	67,86(57,14-78,57)a	58,33(45,83-70,83)a	66,67(50,00-75,00)	56,25(46,88-65,63)
Sim, de forma indireta	64,29(50,00-75,00)b	58,33(45,83-66,67)a	58,33(50,00-75,00)	56,25(46,88-65,63)
Não	67,86(53,57-78,57)a	62,50(45,83-70,83)b	58,33(50,00-75,00)	56,25(46,88-65,63)
**Teve COVID-19**	*			*
Não	67,86(53,57-78,57)	58,33(45,83-70,83)	66,67(50,00-75,00)	56,25(46,88-65,63)
Sim	64,29(46,43-75,00)	58,33(41,67-70,83)	66,67(50,00-75,00)	50,00(40,63-68,75)
Diagnóstico de COVID, sem internação	*	*		
Não	67,86(53,57-78,57)	58,33(45,83-70,83)	66,67(50,00-75,00)	56,25(46,88-65,63)
Sim	64,29(50,00-78,57)	58,33(41,67-70,83)	66,67(50,00-66,67)	50,00(37,50-68,75)
Diagnóstico de COVID, com internação				
Não	67,86(53,57-78,57)	58,33(45,83-70,83)	66,67(50,00-75,00)	56,25(46,88-65,63)
Sim	67,86(53,57-78,57)	58,33(45,83-70,83)	66,67(50,00-75,00)	56,25(46,88-65,63)
Afastamento do trabalho *				*
Não	67,86(57,14-78,57)	58,33(45,83-70,83)	66,67(50,00-75,00)	59,38(46,88-65,63)
Sim	66,07(53,57-75,00)	58,33(45,83-70,83)	66,67(50,00-75,00)	56,25(46,88-65,63)
**Motivo do afastamento**				
Decisão institucional				
Não	64,29(53,57-75,00)	58,33(45,83-70,83)	58,33(50,00-75,00)	56,25(46,88-65,63)
Sim	64,29(53,57-75,00)	58,33(45,83-70,83)	58,33(50,00-75,00)	56,25(46,88-65,63)
Doença crônica	*			
Não	67,86(53,57-78,57)	58,33(45,83-70,83)	66,67(50,00-75,00)	56,25(46,88-65,63)
Sim	64,29(46,43-78,57)	58,33(41,67-70,83)	66,67(50,00-75,00)	56,25(46,88-65,63)
Aspectos psicoemocionais *				*
Não	67,86(53,57-78,57)	58,33(45,83-70,83)	66,67(50,00-75,00)	56,25(46,88-65,63)
Sim	57,14(35,71-71,43)	58,33(37,50-66,67)	66,67(41,67-75,00)	50,00(43,75-65,63)
Escolha pessoal	*	*		
Não	67,86(53,57-78,57)	58,33(45,83-70,83)	66,67(50,00-75,00)	56,25(46,88-65,63)
Sim	69,64(60,71-78,57)	62,50(50,00-70,83)	66,67(50,00-75,00)	56,25(46,88-65,63)
Sintomas gripais	*		*	
Não	67,86(53,57-78,57)	58,33(45,83-70,83)	66,67(50,00-75,00)	56,25(46,88-65,63)
Sim	64,29(50,00-78,57)	58,33(45,83-70,83)	62,50(41,67-75,00)	56,25(43,75-64,06)
Diagnóstico de COVID, sem internação *				*
Não	67,86(53,57-78,57)	58,33(45,83-70,83)	66,67(50,00-75,00)	56,25(46,88-65,63)
Sim	64,29(50,00-78,57)	58,33(41,67-70,83)	66,67(50,00-66,67)	50,00(37,50-68,75)
Diagnóstico de COVID, com internação				
Não	67,86(53,57-78,57)	58,33(45,83-70,83)	66,67(50,00-75,00)	56,25(46,88-65,63)
Sim	67,86(53,57-78,57)	58,33(45,83-70,83)	66,67(50,00-75,00)	56,25(46,88-65,63)

Dados da Tabela 4 apresentados em mediana (Intervalo interquartil: P25 -P75)

* p<0,05, obtido pelo teste de Mann-Whitney (2 grupos) e Kruskal-Wallis (mais de dois grupos). Letras minúsculas diferentes, ladeadas de suas medianas e intervalos interquartis, representam diferenças pelo teste de Dunn

## DISCUSSÃO

A presente pesquisa propõe avaliar a QV dos fonoaudiólogos brasileiros no contexto pandêmico de COVID-19. Algumas pesquisas sobre segurança laboral, saúde mental e QV dos profissionais de saúde durante a pandemia da COVID-19 puderam ser encontradas^(13,16)^.

Um estudo australiano^(17)^ apontou maior risco de abalo psicológico nos profissionais de saúde jovens durante surtos virais, devido às restrições impostas pela quarentena, estigma social e sono comprometido, corroborando o presente estudo no qual o fonoaudiólogo com idade avançada apresentou maiores escores de QV para os domínios psicológico e relações sociais, demonstrando que com avanço da idade a rede social de apoio desse profissional pode melhorar sua QV.

O sexo feminino apresentou pior pontuação em comparação ao masculino nos aspectos físico e psicológico corroborando as pesquisas brasileiras^(18)^ que descreveram escores reduzidos de QV em mulheres estudantes de medicina, nos mesmos domínios do WHOQOL-bref, antes da pandemia.

A maioria dos fonoaudiólogos brasileiros participantes do presente estudo são mulheres e a literatura recente^(19)^ têm evidenciado maior prevalência de sintomatologia para estresse, ansiedade e depressão na população feminina durante a pandemia da COVID-19, além de sua vulnerabilidade emocional vinculada às alterações hormonais durante o período pré-menstrual, pré e pós parto e menopausa, bem como as desigualdades de gênero, as quais fortalecem sobrecargas trabalhistas refletindo na QV.

Fonoaudiólogos pardos e pretos apresentaram menor escore de QV para o domínio meio ambiente. Conforme pesquisa publicada pelo Instituto Brasileiro de Geografia e Estatística, pessoas de pele preta ou parda têm renda inferior mesmo em função semelhante e alcance limitado à cargo superior em comparação aos brancos^(20)^, presume-se que no cenário pandêmico estas discrepâncias afetam ainda mais a QV.

Quanto ao estado civil, fonoaudiólogos casados ou em união estável apresentaram maiores escores de QV para os domínios relações sociais, psicológico e meio ambiente em comparação com os que declararam não ter companheiro, sendo que, estudos demonstram a importância da rede de apoio^(17,19)^ durante a pandemia e *m*ostram o reflexo da convivência social na QV, assim como possível segurança econômica maior quando se pode contar com o cônjuge na gestão das despesas domésticas^(21)^.

Para o domínio meio ambiente, fonoaudiólogos da 7ª região do Conselho de Fonoaudiologia (Rio Grande do Sul) apresentaram melhor escore de QV e a menor pontuação adveio da 9ª região (Acre, Amapá, Amazonas, Pará, Rondônia, Roraima), podendo haver relação com as desigualdades socioeconômicas (na infraestrutura, distribuição de renda, saneamento básico)^(20)^ e com a diferença média de 52% no índice de mortalidade por SARS-COV-2 entre as regiões sul e norte do Brasil (44,1 e 84,4 óbitos para cada 100 mil habitantes, respectivamente)^(7)^, no período de realização desta pesquisa.

Concernente ao ambiente de trabalho, os escores de QV dos fonoaudiólogos em clínica particular, centro de reabilitação, gestão/ administrativo mostraram-se superiores ao dos que atuam em ambulatório, domicílio, unidade de terapia intensiva e cuidado paliativo, possivelmente, conforme apontam outros estudos^(16,22)^ devido ao serviço intenso, a baixa disponibilidade dos equipamentos de proteção individual e a constante sensação de insegurança experimentada pelos profissionais no atendimento hospitalar e/ou domiciliar, como também a suspensão do serviço ambulatorial presencial^(10)^ vinculado ao afastamento do trabalho ou ao teleatendimento, ambos com provável impacto salarial.

Mediante os dados encontrados e as realidades vividas, por nós fonoaudiólogos durante o primeiro período da pandemia, sugere-se aumento da rede de apoio social refletindo no relacionamento cordial e ético entre os colegas bem como a busca por uma rotina de alimentação, prática de exercícios físicos, períodos regulares de sono, terapias, meditações e técnicas de relaxamento como possibilidades no enfrentamento da pandemia COVID-19.

Este estudo apresentou como limitação a elaboração de perguntas mais assertivas quanto aos aspectos profissiográficos. Foram utilizadas estratégias como solicitação da região do conselho e comparação com o estado de residência para restringir as respostas apenas aos fonoaudiólogos, preenchimento único por cada participante e obrigatoriedade nas questões relevantes e complementares, na tentativa de reduzir incertezas às respostas enviadas.

## CONCLUSÃO

O fonoaudiólogo brasileiro apresentou mais baixos escores de QV nos domínios psicológico e meio ambiente no contexto de pandemia da COVID-19. Menores pontuações no domínio físico da QV foram dadas por fonoaudiólogos do sexo feminino, que faziam atendimento ambulatorial, não trabalhavam em clínica particular, atuavam de forma indireta na pandemia, que se afastaram do trabalho e cujo motivo de afastamento foram aspectos psicoemocionais. Já para o domínio psicológico da QV o sexo feminino, a ausência de companheiro, os profissionais que trabalhavam em clínica particular ou em outro local e que aturam de forma direta ou indireta na pandemia apresentaram menores escores em relação aos seus pares.

Na avaliação do domínio relações sociais, os menores escores foram encontrados nos profissionais que não tinham companheiro, naqueles que realizavam atendimento ambulatorial, mas não trabalhavam em clínicas particulares. E por fim, para o domínio meio ambiente, menores escores foram achados na raça/cor preta e parda, nos com ausência de companheiro, nos que faziam atendimento domiciliar, mas não nos que atendiam em clínica particular ou que atuavam na gestão/administrativo ou outro local. Dessa forma, conclui-se que a QV de fonoaudiólogos durante a pandemia está relacionada a fatores sociodemográficos, profissiográficos e relativos à COVID-19.

O fonoaudiólogo brasileiro trabalha na linha de frente para conter a propagação do novo coronavírus e cuidar das pessoas doentes, enquanto coloca a própria vida em risco. Possivelmente a preocupação com o outro sobreponha àquela dispensada a si mesmo, por isso, saber que a família está segura, os amigos e a sociedade valorizam seu trabalho é fundamental para que consigam enfrentar com coragem e esperança a difícil tarefa em que estão empenhados. Estudos apontam que o uso equilibrado das redes sociais, o cuidado pessoal e a empatia são meios de amenizar os impactos na qualidade de vida.

Finalmente, sugere-se mais estudos na área a fim de apontar, associar e comparar a qualidade de vida dos profissionais da saúde nos anos subsequentes à pandemia do novo coronavírus.
